# Clinical and microbiologic characteristics of cefotaxime-non-susceptible *Enterobacteriaceae* bacteremia: a case control study

**DOI:** 10.1186/s12879-016-2150-6

**Published:** 2017-01-07

**Authors:** Taro Noguchi, Yasufumi Matsumura, Masaki Yamamoto, Miki Nagao, Shunji Takakura, Satoshi Ichiyama

**Affiliations:** Department of Clinical Laboratory Medicine, Kyoto University Graduate School of Medicine, 54 Shogoin-Kawahara-cho, Sakyo-ku, Kyoto, 606-8507 Japan

**Keywords:** *Enterobacteriaceae*, Cefotaxime-non-susceptible, Bacteremia

## Abstract

**Background:**

Cefotaxime plays an important role in the treatment of patients with bacteremia due to *Enterobacteriaceae*, although cefotaxime resistance is reported to be increasing in association with extended-spectrum β-lactamase (ESBL) and AmpC β-lactamase (AmpC).

**Methods:**

We conducted a case-control study in a Japanese university hospital between 2011 and 2012. We assessed the risk factors and clinical outcomes of bacteremia due to cefotaxime-non-susceptible *Enterobacteriaceae* (CTXNS-En) and analyzed the resistance mechanisms.

**Results:**

Of 316 patients with *Enterobacteriaceae* bacteremia, 37 patients with bacteremia caused by CTXNS-En were matched to 74 patients who had bacteremia caused by cefotaxime-susceptible *Enterobacteriaceae* (CTXS-En). The most common CTXNS-En was *Escherichia coli* (43%), followed by *Enterobacter* spp. (24%) and *Klebsiella* spp. (22%). Independent risk factors for CTXNS-En bacteremia included previous infection or colonization of CTXNS-En, cardiac disease, the presence of intravascular catheter and prior surgery within 30 days. Patients with CTXNS-En bacteremia were less likely to receive appropriate empirical therapy and to achieve a complete response at 72 h than patients with CTXS-En bacteremia. Mortality was comparable between CTXNS-En and CTXS-En patients (5 vs. 3%). CTXNS-En isolates exhibited multidrug resistance but remained highly susceptible to amikacin and meropenem. CTX-M-type ESBLs accounted for 76% of the β-lactamase genes responsible for CTXNS *E. coli* and *Klebsiella* spp. isolates, followed by plasmid-mediated AmpC (12%). Chromosomal AmpC was responsible for 89% of CTXNS *Enterobacter* spp. isolates.

**Conclusions:**

CTXNS-En isolates harboring ESBL and AmpC caused delays in appropriate therapy among bacteremic patients. Risk factors and antibiograms may improve the selection of appropriate therapy for CTXNS-En bacteremia. Prevalent mechanisms of resistance in CTXNS-En were ESBL and chromosomal AmpC.

**Electronic supplementary material:**

The online version of this article (doi:10.1186/s12879-016-2150-6) contains supplementary material, which is available to authorized users.

## Background

Third-generation cephalosporins, such as cefotaxime, form an important part of empirical antimicrobial therapy for infections caused by members of the *Enterobacteriaceae* family, such as *Escherichia coli* and *Klebsiella pneumoniae*. Third-generation cephalosporins can be a reasonable choice even for patients with nosocomial infections who have non-severe illness. However, a recent increase in the prevalence of third-generation cephalosporin-resistance has challenged the use of this therapy [1]. β-Lactamases have been recognized as the main cause of cephalosporin resistance among *Enterobacteriaceae*. The most common β-lactamases are extended-spectrum β-lactamases (ESBLs), followed by AmpC β-lactamases [2].

When gram-negative bacteria is grown in blood culture, the type of positive blood culture bottle (aerobic or anaerobic) and the gram stain findings help us to estimate if the bacteria belongs to the *Enterobacteriaceae* family or are non-fermenting gram-negative bacteria. It is impossible to determine the exact genus or species without emerging rapid identification technologies, such as matrix-assisted laser desorption-ionization time-of flight mass spectrometry. Therefore, we usually determine a regimen of empiric therapy targeting *Enterobacteriaceae*, not specific species (e.g., *E. coli*). However, most studies regarding bacteremia due to third-generation cephalosporin-non-susceptible *Enterobacteriaceae* have focused on *E. coli* and *K. pneumoniae* [2–5]. A few studies have investigated *Enterobacteriaceae* as a group. Rottier et al. assessed risk factors for bacteremia by third-generation cephalosporin-non-susceptible *Enterobacteriaceae* [6], and two studies analyzed resistance mechanisms [7, 8]. Data from these bacteremias from Japan have not been reported. We conducted this study to determine the risk factors and clinical outcomes associated with bacteremia due to cefotaxime-non-susceptible *Enterobacteriaceae* (CTXNS-En). In addition, we elucidated the epidemiology of β-lactamases that confer resistance to CTXNS-En.

## Methods

### Setting and study design

**Fig. 1:**
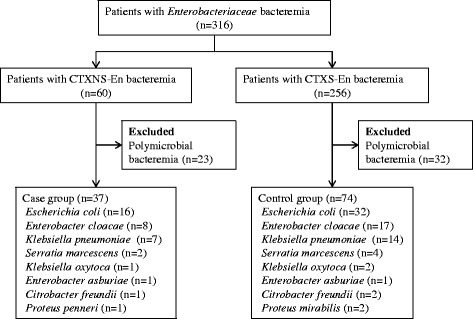
Flow diagram of patient selection process. Three control patients for case patients with *E. asburiae* (*n* = 1) and *P. penneri* (*n* = 2) were not found. Patients infected with organism belonging to the same genus were selected as control patients (*E. cloacae*, *n* = 1; *P. mirabilis*, *n* = 2)

This study was conducted at Kyoto University Hospital, a tertiary care 1182-bed university hospital located in Japan. All patients with bacteremia due to CTXNS-En that occurred from January 2011 to December 2012 were enrolled in this study. Only the first episode of bacteremia was included for each patient. Case patients were defined as adult patients (≥18 years old) with *Enterobacteriaceae* isolates non-susceptible to cefotaxime grown in blood culture. Bacteremia with multiple pathogens was excluded. Control patients were matched in a 1:2 ratio to case patients according to the following algorithm: an adult patient with bacteremia due to cefotaxime-susceptible *Enterobacteriaceae* (CTXS-En) and the infective organism matched to that of the case patient (Fig. 1). If no matched organism was found, the organism that belonged to the same genus was selected. We did not perform routine screening for CTXNS-En colonization*.*

### Definitions and variables

CTXNS-En isolates with minimum inhibitory concentrations (MICs) of >8 μg/mL were defined as CTXNS-En, and isolates with MICs ≤8 μg/mL were considered CTXS-En according to the Clinical and Laboratory Standards Institute (CLSI) guideline (M100-S19) [9]. Each patient was classified as hospital-acquired, health care-associated, or community-acquired in accordance with the definitions of Friedman et al. [10]. Neutropenia was defined as an absolute neutrophil count below 500/mm^3^. Multidrug-resistant-*Enterobacteriaceae* (MDR-En) were defined as *Enterobacteriaceae* with resistance to 3 or more different classes of antibiotics [11].

Clinical characteristics included age, sex, underlying chronic disease, the Charlson weighted index of comorbidity [12], immunosuppressive therapy during the previous 30 days, antibiotic therapy during the previous 30 days, surgery during the previous 30 days, neutropenia, the presence of an intravenous catheter or any other artificial devices, the site of infection, the Sequential Organ Failure Assessment (SOFA) score [13], the site of acquisition, and the antimicrobial regimen and clinical outcomes, including mortality at 30 days.

Antimicrobial therapy was classified into initial empirical and definitive therapy, with the former defined as initial therapy provided within 72 h after bacteremia onset and the latter defined as therapy provided after the results of susceptibility tests had been reported. Antimicrobial therapy was considered appropriate when the isolate was reported as being susceptible to the agent by the clinical microbiology laboratory.

Clinical outcomes were evaluated daily until 7 days after starting antimicrobial therapy and were classified as follows: ‘complete response’ for patients who had resolved fever, leukocytosis and all signs of infection, ‘partial response’ for patients who had abatement of abnormalities in the above parameters without complete resolution and ‘failure’ for patients who had absence of abatement or who had deterioration in any clinical parameters or who died.

### Microbiological analysis

Blood cultures were incubated on the BacT/Alert system (bioMérieux, Marcy l’Etoile, France) for 5 days. When growth was detected, the sample was subcultured and an isolated colony was used in the subsequent processes. All isolates and their antibiotic susceptibilities were determined using the MicroScan WalkAway 96 plus system (Siemens, Berlin, Germany). ESBL screening was performed according to the CLSI microdilution methodology, with modifications (cefpodoxime, ≥4 μg/mL; cefotaxime, ≥8 μg/mL; ceftazidime, ≥2 μg/mL; or aztreonam, ≥8 μg/mL). Quality control was performed using *E. coli* ATCC 25922, ATCC 35218 and *Pseudomonas aeruginosa* ATCC 27853 according to the CLSI document [9]. The confirmation of ESBL production was performed using cefotaxime-clavulanate and ceftazidime-clavulanate disks according to the CLSI guideline [9]. The cefoxitin-cloxacillin disk method was performed to test for chromosoma﻿l AmpC β-lactamase (c-AmpC) hyperproduction. Disks containing 30 μg of cefoxitin or 30 μg of cefoxitin plus 200 μg of cloxacillin were placed on Mueller-Hinton agar that was inoculated with each isolate, and the specimens were incubated at 37 °C for 16 to 18 h. A difference in the inhibition zones of cefoxitin and cloxacillin compared with cefoxitin alone of ≥4 mm was considered to be indicative of c-AmpC hyperproduction [14].

Bacterial DNA was isolated using a QIAamp DNA Mini kit (Qiagen, Hilden, Germany). PCR analyses for the detection of TEM-, SHV-, and CTX-M-type β-lactamase genes were conducted as described elsewhere [15–19]. Plasmid-mediated AmpC β-lactamase (p-AmpC) was detected using the multiplex PCR method [20] and was identified by sequencing. Isolates negative for ESBL and p-AmpC by PCR were then tested using the cefoxitin-cloxacillin disk method, as described above. Isolates non-susceptible to imipenem or meropenem (MIC > 1 μg/mL) were analysed to determine the presence of the carbapenemases using multiplex PCR [15]. Primers used for PCR and sequencing is provided in Additional file 1: Table S1.

### Statistical analysis

Comparisons of discrete variables were performed with Fisher’s exact test, and comparisons of continuous variables were performed using Wilcoxon–Mann–Whitney tests. The time to a complete response within 7 days after starting antimicrobial therapy was analyzed using a Cox-hazard model. To determine the independent risk factors for CTXNS-En bacteremia and the time to a complete response, all variables with *p*-values of < 0.05 based on univariate analyses were subjected to further selection using forward stepwise logistic regression. We forced the inclusion of the Charlson index, the SOFA score and CTXNS-En bacteremia in the multivariate models. A *p*-value of < 0.05 was considered to be statistically significant. The statistical analysis was performed using STATA software version 13 (StataCorporation College Station, TX, USA).

## Results

### Risk factors and clinical outcomes

A total of 316 non-duplicate patients with *Enterobacteriaceae* bacteremia were identified during the study period. Of these, 60 patients (19%) had infections caused by isolates that showed non-susceptibility to cefotaxime. Patients with polymicrobial bacteremia (*n* = 23) were excluded from the analysis, and 37 patients were analyzed as the CTXNS-En group. Of the remaining 256 patients with CTXS-En bacteremia, 32 patients with polymicrobial bacteremia were excluded, and 74 patients were selected as controls. Patients selection process and bacterial species were shown in Fig. 1.

The demographics of the patients and risk factors associated with bacteremia due to CTXNS-En and CTXS-En are listed in Table 1. The distributions for age and sex, severities of illness as measured by SOFA score, and the location of acquisition were comparable. Primary bacteremia, urinary tract infection, and intraabdominal infection were common sources of infection in both groups.

**Table 1:** Univariate and multivariate analyses of risk factors for patients with CTXNS-En and CTXS-En bacteremia

Characteristics	CTXNS-En (*n* = 37)	CTXS-En (*n* = 74)	Univariate analysis		Multivariate analysis	
			OR (95% CI)	*P*-value	OR (95% CI)	*P*-value
Age	66 (19–93)	67 (19–90)		0.98		
Male	24 (65%)	41 (55%)		0.34		
Nosocomial or healthcare-associated bacteremia	34 (92%)	59 (80%)	2.88 (0.73–16.49)	0.17		
Previous isolation of MDR bacteria	15 (41%)	9 (12%)	4.92 (1.71–14.51)	<0.01		
Previous isolation of CTXNS-En	17 (46%)	6 (8%)	9.63 (3.04–33.20)	<0.01	12.32 (3.69–41.12)	<0.01
Previous isolation of fluoroquinolone-resistant *Enterobacteriaceae*	11 (30%)	7 (9%)	4.05 (1.26–13.58)	0.01		
Previous ICU admission within 30 days	9 (24%)	11 (15%)	1.84 (0.60–5.50)	0.29		
Previous antibiotic use within 30 days^a^						
Any antibiotics	28 (76%)	38 (51%)	2.95 (1.14–8.04)	0.02		
Penicillins	2 (5%)	2 (3%)	2.06 (0.14–29.27)	0.60		
Third-generation cephalosporins	8 (22%)	7 (9%)	2.64 (0.75–9.36)	0.09		
Other cephems	15 (41%)	14 (19%)	2.92 (1.11–7.69)	0.02		
β-lactam/β-lactamase inhibitors	6 (16%)	3 (4%)	4.58 (0.90–29.66)	0.06		
Carbapenems	6 (16%)	5 (7%)	2.67 (0.62–11.85)	0.18		
Fluoroquinolones	6 (16%)	7 (9%)	1.85 (0.47–7.01)	0.35		
Aminoglycosides	0 (0%)	1 (1%)	0	1.00		
Trimethoprim/sulfamethoxazole	8 (22%)	14 (19%)	1.18 (0.38–3.43)	0.80		
Glycopeptides	7 (19%)	6 (8%)	2.61 (0.68–10.17)	0.12		
Duration of previous antibiotic use	11 (0–30)	7 (0–30)		0.02		
Charlson index	4 (0–9)	3 (0–14)		0.21		
Hematological malignancy	7 (19%)	7 (9%)	2.23 (0.60–8.14)	0.22		
Solid malignancy	11 (30%)	30 (41%)	0.62 (0.24–1.55)	0.30		
Transplantation	10 (27%)	6 (8%)	4.20 (1.22–15.31)	0.01		
Hemodialysis	4 (11%)	5 (7%)	1.67 (0.31–8.30)	0.48		
Diabetes	9 (24%)	19 (26%)	0.93 (0.33–2.51)	1.00		
Cardiac disease	18 (49%)	16 (22%)	3.43 (1.35–8.75)	<0.01	5.00 (1.64–15.28)	<0.01
Chronic lung disease	8 (22%)	12 (16%)	1.43 (0.45–4.27)	0.60		
Liver disease	10 (27%)	17 (23%)	1.24 (0.44–3.33)	0.65		
Pancreatobilliary disease	4 (11%)	20 (27%)	0.33 (0.08–1.11)	0.05		
Chronic kidney disease	13 (35%)	27 (36%)	0.94 (0.38–2.31)	1.00		
Connective tissue disease	4 (11%)	8 (11%)	1.00 (0.21–4.06)	1.00		
Intravascular catheter	31 (84%)	40 (54%)	4.39 (1.54–14.26)	<0.01	5.33 (1.46–19.49)	0.01
Urinary catheter	16 (43%)	19 (26%)	2.21 (0.88–5.50)	0.08		
Mechanical ventilation	6 (16%)	4 (5%)	3.39 (0.73–17.31)	0.08		
Other artificial devices	19 (51%)	22 (30%)	2.49 (1.02–6.10)	0.04		
Use of immunosuppressive therapy within 30 days	20 (54%)	40 (54%)	1.00 (0.42–2.39)	1.00		
Neutropenia	5 (14%)	12 (16%)	0.807 (0.20–2.74)	0.79		
Previous surgery within 30 days^b^	12 (32%)	6 (8%)	5.44 (1.65–19.34)	<0.01	4.37 (1.17–16.41)	0.03
Invasive procedure within 30 days	17 (46%)	18 (24%)	2.64 (1.05–6.63)	<0.03		
Source of infection						
Urinary tract infection	8 (22%)	16 (22%)	1.00 (0.33–2.83)	1.00		
Intraabdominal infection	7 (19%)	18 (24%)	0.73 (0.23–2.09)	0.63		
Pneumonia	2 (5%)	2 (3%)	2.06 (0.14–29.27)	0.60		
Primary bacteremia	14 (38%)	26 (35%)	1.12 (0.45–2.74)	0.84		
Other infection	6 (16%)	12 (16%)	1.00 (0.28–3.22)	1.00		
SOFA score	3 (0–10)	3 (0–21)		0.34		

Data are presented as nos. (%) or medians (interquartile ranges)

*MDR* multidrug-resistant, *OR* odds ratio, *CI* confidence interval

^a^All 10 patients who had bacteremia with c-AmpC-overproducing *Enterobacteriaceae,* including 8 *E. cloacae*, 1 *E. asbriae* and 1 *C. freundii*, were exposed to β-lactams within 30 days

^b^The numbers of patients who had cardiovascular surgery within 30 days were similar in the CTXNS-En and CTXS-En groups (*n* = 1 [3%] and *n* = 3 [4%], respectively; *p* = 0.72)

A risk factor analysis for the CTXNS-En revealed significant associations in the univariate analysis with a previous infection or colonization of CTXNS-En or MDR bacteria or fluoroquinolone-resistant *Enterobacteriaceae*, previous antibiotic use, duration of previous antibiotic therapy, cardiac disease, transplantation and prior surgery. In the multivariate analysis, previous infection or colonization of CTXNS-En (odds ratio [OR], 12.32; 95% confidence interval [CI], 3.69–41.12), cardiac disease (OR, 5.00; CI, 1.64–15.28), the presence of an intravascular catheter (OR, 5.33; CI, 1.46–19.49) and prior surgery within 30 days (OR, 4.37; CI, 1.17–16.41) were independent risk factors for CTXNS-En bacteremia.

Complete response was achieved within 72 h in 4 (11%) and 23 (31%) of the patients in CTXNS-En and CTXS-En groups, respectively (Table 2). The CTXNS-En patients were less likely to receive appropriate empirical antimicrobial therapy than the CTXS-En patients. Mortality at 30 days was low in both the CTXNS-En and CTXS-En groups (5 and 3%, respectively).

**Table 2:** Univariate analysis of the treatments and outcomes of patients with CTXNS-En and CTXS-En bacteremia

Characteristics	CTXNS-En^a^ (*n* = 37)	CTXS-En (*n* = 74)	*P*-value
Empirical therapy			
Third-generation cephalosporins	5 (14%)	20 (27%)	0.15
Other cephems	10 (27%)	21 (28%)	1.00
β-lactam/β-lactamase inhibitors	11 (30%)	15 (20%)	0.34
Carbapenems	10 (27%)	16 (22%)	0.64
Other antibiotics^b^	0 (0%)	2 (3%)	1.00
Appropriate empirical therapy	23 (62%)	66 (89%)	<0.01
Complete response at 72 h	4 (11%)	23 (31%)	0.02
Complete response at 7 days	23 (62%)	59 (80%)	0.07
30-day mortality	2 (5%)	2 (3%)	0.60

^a^One patient died before antibiotics could be administered

^b^Other antibiotics included amikacin (*n* = 1) and levofloxacin (*n* = 1)

Univariate analysis using the Cox-hazard model revealed that the variables independently associated with complete response were the presence of an intravascular catheter, the SOFA score, empirical carbapenem therapy and empirical third-generation cephalosporin therapy (Table 3). In the multivariate analysis, the SOFA score (hazard ratio [HR], 0.88; CI, 0.80–0.96) and empirical third-generation cephalosporin therapy (HR, 2.13; CI, 1.30–3.50) were significant predictors of a complete response.

**Table 3:** Predictors of complete response within 7 days of 111 patients with CTXNS-En and CTXS-En bacteremia

Characteristics	Univariate analysis		Multivariate analysis	
	HR (95% CI)	*P*-value	HR (95% CI)	*P*-value
Age	1.01 (0.99–1.02)	0.26		
Male	1.09 (0.70–1.70)	0.70		
Nosocomial or healthcare-associated bacteremia	0.85 (0.48–1.51)	0.58		
Charlson index	0.94 (0.85–1.03)	0.20	0.98 (0.89–0.96)	0.74
Hematological malignancy	1.03 (0.53–2.00)	0.93		
Solid malignancy	0.95 (0.60–1.49)	0.82		
Transplantation	0.65 (0.33–1.30)	0.23		
Hemodialysis	0.43 (0.16–1.19)	0.10		
Diabetes	1.07 (0.65–1.76)	0.79		
Cardiac disease	0.86 (0.54–1.37)	0.52		
Chronic lung disease	0.99 (0.56–1.73)	0.96		
Liver disease	0.69 (0.40–1.19)	0.18		
Pancreatobilliary disease	0.93 (0.55–1.60)	0.81		
Chronic kidney disease	0.95 (0.60–1.50)	0.83		
Connective tissue disease	0.87 (0.42–1.81)	0.72		
Intravascular catheter	0.60 (0.39–0.93)	0.02		
Urinary catheter	0.69 (0.42–1.13)	0.14		
Mechanical ventilation	0.50 (0.20–1.24)	0.13		
Other artificial devices	0.74 (0.46–1.17)	0.20		
Use of all immunosuppressive therapy within 30 days	0.90 (0.58–1.39)	0.64		
Neutropenia	0.88 (0.47–1.66)	0.70		
Previous surgery within 30 days	0.69 (0.37–1.27)	0.23		
Invasive procedure within 30 days	0.63 (0.39–1.04)	0.07		
Source of infection				
Urinary tract infection	1.17 (0.71–1.94)	0.54		
Intraabdominal infection	0.72 (0.42–1.23)	0.23		
Pneumonia	0.74 (0.23–2.36)	0.62		
Primary bacteremia	0.85 (0.53–1.36)	0.50		
SOFA score	0.86 (0.79–0.94)	<0.01	0.88 (0.80–0.96)	<0.01
Empirical therapy				
Carbapenems	0.41 (0.22–0.75)	<0.01		
Third-generation cephalosporins^a^	2.54 (1.56–4.14)	<0.01	2.13 (1.30–3.50)	<0.01
Other cephems	1.32 (0.83–2.10)	0.25		
β-lactam/β-lactamase inhibitors	0.66 (0.39–1.11)	0.12		
Appropriate empirical therapy	1.43 (0.79–2.58)	0.24		
CTXNS-En bacteremia	0.64 (0.39–1.03)	0.07	0.73 (0.45–1.19)	0.20

Data are presented as nos. (%) or medians (interquartile ranges)

One patient with CTXNS-En bacteremia and 2 patients with CTXS-En bacteremia were excluded from the analysis because they died before the achievement of complete response at 30 days

All variables with *p*-values less than 0.05 in the univariate analyses were included in the multivariate analysis. Stepwise logistic regression analysis was performed using forward selection and the likelihood ratio. The Charlson index, SOFA score and CTXNS-En bacteremia were forced into the models

*MDR* multidrug-resistant, *HR* hazard ratio, *CI* confidence interval

^a^Patients who received third-generation cephalosporin as empirical therapy had lower SOFA scores compared with patients who received other empirical therapies (median = 2 [range: 0–9] and median = 3 [range: 0–21], respectively; *p* < 0.01)

### Microbiological results

The susceptibility testing results for the CTXNS-En and CTXS-En isolates of the case and control groups are shown in Table 4. Amikacin and meropenem were highly active, with greater than 90% of CTXNS-En isolates being susceptible to them. Susceptibility to cephalosporins, β-lactam/β-lactamase inhibitor combinations, fluoroquinolones and sulfamethoxazole-trimethoprim was uncommon in CTXNS-En isolates compared with CTXS-En isolates, and CTXNS-En isolates more frequently exhibited multidrug resistance than CTXS-En isolates. Although one *K. pneumoniae* isolate and one *K. oxytoca* isolate had meropenem MICs of > 1 μg/mL, the multiplex PCR did not detect the presence of carbapenemases.

**Table 4:** Antimicrobial susceptibility of 111 *Enterobacteriaceae* isolates recovered from patients with CTXNS-En and CTXS-En bacteremia

Antimicrobial agent	No. (%)		*P*-value
	CTXNS-En (*n* = 37)	CTXS-En (*n* = 74)	
Ceftazidime	5 (14%)	73 (99%)	<0.01
Cefozopran	12 (32%)	73 (99%)	<0.01
Cefepime	21 (57%)	70 (95%)	<0.01
Flomoxef^a^	22 (59%)	56 (76%)	0.12
Meropenem	36 (97%)	74 (100%)	<0.33
Amoxicillin-clavulanate	13 (35%)	46 (62%)	<0.01
Piperacillin-tazobactam	25 (68%)	74 (100%)	<0.01
Amikacin	37 (100%)	74 (100%)	1.00
Gentamicin	28 (76%)	64 (86%)	0.18
Ciprofloxacin	18 (49%)	65 (88%)	<0.01
Levofloxacin	19 (51%)	65 (88%)	<0.01
Sulfamethoxazole**-**trimethoprim	20 (54%)	57 (77%)	0.02
Multidrug resistance	26 (70%)	10 (14%)	<0.01

The CLSI breakpoints (M100-S19) were used as interpretive criteria

^a^Isolates were regarded as susceptible to flomoxef at an MIC of ≤ 8 μg/mL

ESBL production was confirmed in 20 isolates among 37 CTXNS-En isolates, 19 of which harbored ESBL gene. All CTXS-En isolates were negative for ESBL confirmation test. The types of β-lactamase genes for the CTXNS-En isolates from the case group are shown in Table 5. The most prevalent β-lactamase gene harbored by *E. coli* was ESBL. In *E. coli*, *bla*_CTX-M-14_ was the most prevalent gene detected, followed by *bla*_CTX-M-27_. In the *K. pneumoniae* isolates, both ESBL and p-AmpC genes were dominant. *bla*_DHA-1_ was the only p-AmpC detected in *K. pneumoniae*. All but one isolate of *Enterobacter* spp. were considered to overproduce c-AmpC. The resistance mechanisms in *P. penneri* (*n* = 1) and *S. marcescens* (*n* = 2) were not determined.

**Table 5:** Distribution of resistance mechanisms in 37 CTXNS-En isolates

	ESBL					p-AmpC		c-AmpC
Bacterial species	CTX-M-1 group^a^	CTX-M-2 group^b^	CTX-M-9 group^c^	TEM	SHV^d^	CMY-2	DHA-1	
*E. coli* ^e^ (*n* = 16)	2	0	13	0	0	2	0	0
*E. cloacae* ^f^ (*n* = 8)	0	0	0	0	1	0	1	7
*K. pneumoniae* ^g^ (*n* = 7)	0	1	2	0	1	0	3	0
*S. marcescens* (*n* = 2)	0	0	0	0	0	0	0	0
*C. freundii* (*n* = 1)	0	0	0	0	0	0	0	1
*E. asbriae* (*n* = 1)	0	0	0	0	0	0	0	1
*K. oxytoca* (*n* = 1)	0	0	1	0	0	0	0	0
*P. penneri* (*n* = 1)	0	0	0	0	0	0	0	0

^a^ *bla*_CTX-M-15_ (*n* = 1), *bla*_CTX-M-55_ (*n* = 1)

^b^ *bla*_CTX-M-2_ (*n* = 1)

^c^ *bla*_CTX-M-14_ (*n* = 8), *bla*_CTX-M-27_ (*n* = 4), *bla*_CTX-M-9_ (*n* = 1) of *E. coli*, *bla*_CTX-M-14_ (*n* = 2) of *K. pneumoniae* and *bla*_CTX-M-14_ (*n* = 1) of *K. oxytoca*

^d^ *bla*_SHV-12_ (*n* = 1), *bla*_SHV-27_ (*n* = 1)

^e^One isolate carried *bla*_CTX-M-9_ and *bla*_CMY-2_

^f^One isolate carried *bla*_SHV-12_ and *bla*_DHA-1_. The other 7 isolates were positive for the c-AmpC hyperproduction test using the cefoxitin-cloxacillin disk method

^g^One isolate carried *bla*_SHV-27_ and *bla*_CTX-M-14_, and 1 isolate did not carry either ESBL or p-AmpC genes

## Discussion

The prevalence of CTXNS-En varies across different geographic regions. A study from Spain revealed that 9.7, 12.5 and 29.1% of third-generation cephalosporin resistance in bloodstream infections were caused by *E. coli*, *K. pneumoniae* and *Enterobacter* spp., respectively [21]. In the SENTRY program study from the United States of America, the prevalence of third-generation cephalosporin-resistant *Enterobacteriaceae* that caused bacteremia was 6.4% [8]. In the Asia-Pacific region, approximately 10% of *Enterobacteriaceae* were phenotypically positive for ESBL production [22]. The prevalence of CTXNS-En in our study was consistent with these findings.

Previous antibiotic therapy, especially the use of cephalosporins, has been consistently recognized as a risk factor for third-generation cephalosporin-resistant *Enterobacteriaceae* bacteremia in many studies [2, 3, 6, 23]. Our study also demonstrated a significant association between previous antibiotic use and CTXNS-En bacteremia in the univariate analysis. Prior isolation of CTXNS-En also appears to be a potent risk factor [4, 6, 24], as shown in our study. These common risk factors found in both previous studies and our study may help to identify patients with CTXNS-En bacteremia. Although certain risk factors, including cardiovascular disease, the presence of intravascular catheters, and prior surgery within 30 days, suggested cardiovascular surgery as a risk factor for CTXNS-En bacteremia, prior cardiovascular surgery within 30 days was comparable between the CTXNS-En and CTXS-En groups. Patients with intravascular catheters are more likely to be hospitalized patients who receive complicated medical care and are more likely to acquire antibiotic-resistant pathogens. These results suggest that impaired patients who had undergone surgery had a high risk of acquiring CTXNS-En bacteremia.

Patients with bacteremia due to drug-resistant *Enterobacteriaceae* have likely received inappropriate empirical therapy leading to worse mortality [2, 25, 26]. Although mortality was comparable between the CTXNS-En and CTXS-En groups in our study, empirical antimicrobial treatment was more frequently inappropriate among patients with CTXNS-En bacteremia, and a complete response was delayed in the CTXNS-En group compared with the CTXS-En group. Thus, we used a multivariate Cox-hazard model for time to complete response to further assess the association between CTXNS and delayed complete response. However, empirical third-generation cephalosporin therapy was associated with an earlier complete response. This result can be explained by the fact that patients who received third-generation cephalosporin as an empirical therapy had lower SOFA scores, indicating a less severe state of illness, compared with patients who received other empirical therapies (data shown in the footnote of Table 3). Other studies have also found that illness severity appears to be a more significant prognostic factor for clinical outcomes than appropriate antibiotic therapy [3, 27–29]. Nonetheless, the administration of appropriate antibiotic therapy is essential for the successful treatment of bacteremia, especially in patients with severe presentations.

In our study, CTXNS-En isolates had high rates of susceptibility to amikacin and meropenem. Treatments other than cefotaxime, such as amikacin or meropenem, should be considered for patients suspected of *Enterobacteriaceae* bacteremia with risk factors for CTXNS-En. Two isolates had meropenem MICs of greater than 1 μg/mL. Although carbapenems remain the drugs of choice for serious infections caused by *Enterobacteriaceae*, there is concern about the rise of carbapenem resistance in *Enterobacteriaceae* [30]. Balancing the appropriateness of therapy and antibiotic overuse is essential. CTXNS-En isolates were likely to be MDR; co-resistance to fluoroquinolone is increasing in third-generation cephalosporin-resistant *Enterobacteriaceae* [21, 31]. The use of antimicrobial agents will continue to create selection pressure that gives MDR-En the opportunity to become effective intestinal colonizers and provides opportunities for MDR-En to cause infections with limited therapeutic options [30]. Continuous monitoring of MDR-En and antimicrobial stewardship are recommended as important efforts to control MDR-En.

Ninety-four percent of *E. coli* and 43% of *K. pneumoniae* carried CTX-M-type ESBL genes, the majority of which encoded CTX-M-14. Whereas CTX-M-15 is the most widely distributed CTX-M-type ESBL worldwide [32], CTX-M-14, CTX-M-15 and CTX-M-27 are the most prevalent ESBL types of *E. coli* in Asia and Japan [22, 31]. Previous studies have suggested a low prevalence of ESBL-producing *Enterobacter* spp. and *C. freundii* in Japan [33, 34], and only one isolate of these organisms harbored the ESBL gene in our study. Whereas the predominance of CMY-2 has been described worldwide, including in Asia [22, 35], DHA-1 is the most dominant p-AmpC in *K. pneumoniae* isolates in Japan and Asia [22, 36], which is consistent with the findings of our study. Many *Enterobacteriaceae* species, such as *Enterobacter* spp., *Citrobacter* spp. and *S. marcescens,* encode c-AmpC, which can be expressed at high levels by either induction or selection for derepressed mutants in the presence of β-lactam antibiotics [35]. All of the patients who had bacteremia with c-AmpC-overproducing CTXNS-En were exposed to β-lactam within 30 days. The resistance mechanism was not determined in *S. marcescens* and *P. penneri. P. penneri* harbors a class A β-lactamase, HugA, which confers third-generation cephalosporin-resistance and is regulated by an equivalent of the amp system, a regulation system of c-AmpC, although we did not assess HugA β-lactamase [37].

There were some potential limitations to this study. First, the sample size was small, making type II error a concern. Second, our results were from a single center, and the prevalence of CTXNS-En isolates likely varies across institutions, making it difficult to generalize the prevalence of CTXNS-En in our patients to those at other institutions. Third, we used the old CLSI breakpoint for cefotaxime because the treatment decision was made based on the old CLSI breakpoint. The revised CLSI guideline in 2010 adopted a lower breakpoint for cefotaxime (≤1 μg/ml) [38], which may limit the generalizability of this study.

## Conclusions

CTXNS-En bacteremia is associated with inappropriate empirical therapy, and the frequent occurrence of a delay in appropriate therapy likely contributes to inferior clinical responses. CTXNS-En isolates were likely to be MDR. Treatments other than cefotaxime, such as amikacin or meropenem, should be considered for patients suspected of *Enterobacteriaceae* bacteremia with risk factors for CTXNS-En or severe illness. However, cefotaxime may remain the treatment of choice for patients without these risk factors. Acquired β-lactamases, especially CTX-M type ESBL, were common in *E. coli* and *Klebsiella* spp., whereas hyperactivation of intrinsic resistance was common in *Enterobacter* spp.

## Additional file

Additional file 1: Table S1. Primers used for PCR and sequence. (DOCX 28 kb)

## Abbreviations

AmpC: AmpC β-lactamase
c-AmpC: Chromosomal AmpC β-lactamase
CI: Confidence interval
CLSI: Clinical and Laboratory Standards Institute
CTXNS-En: Cefotaxime-non-susceptible *Enterobacteriaceae*
CTXS-En: Cefotaxime-susceptible *Enterobacteriaceae*
ESBL: Extended-spectrum β-lactamase
HR: Hazard ratio
MDR: Multidrug-resistant
MDR-En: MDR-*Enterobacteriaceae*
MICs: Minimum inhibitory concentrations
OR: Odds ratio
p-AmpC: Plasmid-mediated AmpC β-lactamase
SOFA: Sequential Organ Failure Assessment
