# Supplementary material for: Clinical and microbiologic characteristics of cefotaxime-non-susceptible Enterobacteriaceae bacteremia: a case control study
Source: BMC Infect Dis. 2017 Jan 7;17:44. doi: 10.1186/s12879-016-2150-6 (PMC5219717; doi:10.1186/s12879-016-2150-6)
Supplement: Additional file 1: Table S1. — Primers used for PCR and sequence. (DOCX 28 kb) [file 12879_2016_2150_MOESM1_ESM.docx]

Table S1. Primers used for PCR and sequencing

| Target region | Primer name | Primer sequence | Reference |
| --- | --- | --- | --- |
| *bla*_TEM_ | Multi-TSO-T for | CATTTCCGTGTCGCCCTTATTC | [1] |
|  | Multi-TSO-T rev | CGTTCATCCATAGTTGCCTGAC | [1] |
| *bla*_SHV_ | Multi-TSO-S for | AGCCGCTTGAGCAAATTAAAC | [1] |
|  | Multi-TSO-S rev | ATCCCGCAGATAAATCACCAC | [1] |
| *bla*_OXA-1_, *bla*_OXA-4_ and *bla*_OXA-30_ | Multi-TSO-O for | GGCACCAGATTCAACTTTCAAG | [1] |
|  | Multi-TSO-O rev | GACCCCAAGTTTCCTGTAAGTG | [1] |
| *bla*_CTX-M-1_ group | CTXM7 | GCGTGATACCACTTCACCTC | [2] |
|  | CTXM8 | TGAAGTAAGTGACCAGAATC | [2] |
| *bla*_CTX-M-2_ group | CTXM17 | TGATACCACCACGCCGCTC | [2] |
|  | CTXM18 | TATTGCATCAGAAACCGTGGG | [2] |
| *bla*_CTX-M-9_ group | CTXM11 | ATCAAGCCTGCCGATCTGGTTA | [2] |
|  | CTXM12 | GTAAGCTGACGCAACGTCTGC | [2] |
| *bla*_TEM_ (sequencing) | tem-F | TTCTTGAAGACGAAAGGGC | [3] |
|  | tem-R | ACGCTCAGTGGAACGAAAAC | [3] |
| *bla*_SHV_ (sequencing) | shv-F | CACTCAAGGATGTATTGTG | [4] |
|  | shv-R | TTAGCGTTGCCAGTGCTCG | [4] |
| *bla*_CTX-M-1_ group (sequencing) | M13U | GGTTAAAAAATCACTGCGTC | [5] |
|  | M13L | TTGGTGACGATTTTAGCCGC | [5] |
| *bla*_CTX-M-2_ group (sequencing) | M25U | ATGATGACTCAGAGCATTCG | [5] |
|  | M25L | TGGGTTACGATTTTCGCCGC | [5] |
| *bla*_CTX-M-9_ group (sequencing) | M9U | ATGGTGACAAAGAGAGTGCA | [5] |
|  | M9L | CCCTTCGGCGATGATTCTC | [5] |
| *bla*_MOX-1_, *bla*_MOX-2_, *bla*_CMY-1_, *bla*_CMY-8_ to *bla*_CMY-11_ | MOXMF | GCTGCTCAAGGAGCACAGGAT | [6] |
|  | MOXMR | CACATTGACATAGGTGTGGTGC | [6] |
| *bla*_LAT-1_ to *bla*_LAT-4_, *bla*_CMY-2_ to *bla*_CMY-7_, *bla*_BIL-1_ | CITMF | TGGCCAGAACTGACAGGCAAA | [6] |
|  | CITMR | TTTCTCCTGAACGTGGCTGGC | [6] |
| *bla*_DHA-1_, *bla*_DHA-2_ | DHAMF | AACTTTCACAGGTGTGCTGGGT | [6] |
|  | DHAMR | CCGTACGCATACTGGCTTTGC | [6] |
| *bla*_ACC_ | ACCMF | AACAGCCTCAGCAGCCGGTTA | [6] |
|  | ACCMR | TTCGCCGCAATCATCCCTAGC | [6] |
| *bla*_MIR-1T_, *bla*_ACT-1_ | EBCMF | TCGGTAAAGCCGATGTTGCGG | [6] |
|  | EBCMR | CTTCCACTGCGGCTGCCAGTT | [6] |
| *bla*_FOX-1_ to *bla*_FOX-5b_ | FOXMF | AACATGGGGTATCAGGGAGATG | [6] |
|  | FOXMR | CAAAGCGCGTAACCGGATTGG | [6] |
| *bla*_GES-1_ to *bla*_GES-9_ and *bla*_GES-11_ | MultiGES_for | AGTCGGCTAGACCGGAAAG | [1] |
|  | MultiGES_rev | TTTGTCCGTGCTCAGGAT | [1] |
| *bla*_OXA-48-like_ | MultiOXA-48_for | GCTTGATCGCCCTCGATT | [1] |
|  | MultiOXA-48_rev | GATTTGCTCCGTGGCCGAAA | [1] |
| *bla*_IMP_ variants except *bla*_IMP-9_, *bla*_IMP-16_, *bla*_IMP-18_, *bla*_IMP-22_ and *bla*_IMP-25_ | MultiIMP_for | TTGACACTCCATTTACDG | [1] |
|  | MultiIMP_rev | GATYGAGAATTAAGCCACYCT | [1] |
| *bla*_VIM_ variants including *bla*_VIM-1_ and *bla*_VIM-2_ | MultiVIM_for | GATGGTGTTTGGTCGCATA | [1] |
|  | MultiVIM_rev | CGAATGCGCAGCACCAG | [1] |
| *bla*_KPC-1_ to *bla*_KPC-5_ | MultiKPC_for | CATTCAAGGGCTTTCTTGCTGC | [1] |
|  | MultiKPC_rev | ACGACGGCATAGTCATTTGC | [1] |

**References for additional file 1**

1. Dallenne C, Da Costa A, Decré D, Favier C, Arlet G. Development of a set of multiplex PCR assays for the detection of genes encoding important beta-lactamases in Enterobacteriaceae. J Antimicrob Chemother. 2010;65:490-5.

2. Xu L, Ensor V, Gossain S, Nye K, Hawkey P. Rapid and simple detection of blaCTX-M genes by multiplex PCR assay. J Med Microbiol. 2005;54:1183-7.

3. Briñas L, Zarazaga M, Sáenz Y, Ruiz-Larrea F, Torres C. Beta-lactamases in ampicillin-resistant Escherichia coli isolates from foods, humans, and healthy animals. Antimicrob Agents Chemother. 2002;46:3156-63.

4. Yagi T, Kurokawa H, Shibata N, Shibayama K, Arakawa Y. A preliminary survey of extended-spectrum beta-lactamases (ESBLs) in clinical isolates of Klebsiella pneumoniae and Escherichia coli in Japan. FEMS Microbiol Lett. 2000;184:53-6.

5. Saladin M, Cao VT, Lambert T, Donay JL, Herrmann JL, Ould-Hocine Z, et al. Diversity of CTX-M beta-lactamases and their promoter regions from Enterobacteriaceae isolated in three Parisian hospitals. FEMS Microbiol Lett. 2002;209:161-8.

6. Pérez-Pérez FJ, Hanson ND. Detection of plasmid-mediated AmpC beta-lactamase genes in clinical isolates by using multiplex PCR. J Clin Microbiol. 2002;40:2153-62.
